# Single-Atom Fe-Anchored Nano-Diamond With Enhanced Dual-Enzyme Mimicking Performance for H_2_O_2_ and Glutathione Detection

**DOI:** 10.3389/fbioe.2021.790849

**Published:** 2022-01-03

**Authors:** Ying Liu, Jianghong Yan, Yu Huang, Zhiheng Sun, Huijing Zhang, Lihaoyuan Fu, Xuwen Li, Yongri Jin

**Affiliations:** ^1^ College of Chemistry, Jilin University, Changchun, China; ^2^ First Clinical Hospital, Jilin Province Academy of Traditional Chinese Medicine, Changchun, China

**Keywords:** nanodiamond, single-atom Fe, peroxidase-like and oxidase-like activity, colorimetric sensor, glutathione

## Abstract

Glutathione (GSH) is an important antioxidant and free radical scavenger that converts harmful toxins into harmless substances and excretes them out of the body. In the present study, we successfully prepared single-atom iron oxide-nanoparticle (Fe-NP)-modified nanodiamonds (NDs) named Fe-NDs *via* a one-pot *in situ* reduction method. This nanozyme functionally mimics two major enzymes, namely, peroxidase and oxidase. Accordingly, a colorimetric sensing platform was designed to detect hydrogen peroxide (H_2_O_2_) and GSH. Owing to their peroxidase-like activity, Fe-NDs can oxidize colorless 3,3′,5,5′-tetramethylbenzidine (TMB) into blue with sufficient linearity at H_2_O_2_ concentrations of 1–60 μM and with a detection limit of 0.3 μM. Furthermore, using different concentrations of GSH, oxidized TMB can be reduced to TMB, and the color change from blue to nearly colorless can be observed by the naked eye (linear range, 1–25 μM; detection limit, 0.072 μM). The established colorimetric method based on oxidase-like activity can be successfully used to detect reduced GSH in tablets and injections with good selectivity and high sensitivity. The results of this study exhibited reliable consistency with the detection results obtained using high-performance liquid chromatography (HPLC). Therefore, the Fe-NDs colorimetric sensor designed in this study offers adequate accuracy and sensitivity.

## Introduction

Glutathione (GSH), an important tripeptide thiol (γ-glutamyl cysteinyl glycine) antioxidant, is widely found in human cells and involved in several metabolic processes (Richie et al., 1996; Xu et al., 2016; Yan et al., 2016). It plays a significant role in biological systems, including the maintenance of protein structure, intracellular signal transduction, generegulation, and regulation of immune function. Changes in the concentration of GSH are directly associated with the occurrence of some diseases, such as neurodegenerative disorders, inflammation, heart disease, and cancer (Refsum et al., 1998; Zhang et al., 2004; Lu, 2009; Jung et al., 2013; Micke et al., 2015; González de Vega et al., 2016). Studies have shown that glutathione supplementation can prevent some diseases, such as cardiovascular disease, liver disease, diabetes, and delay aging (Micke et al., 2015; González de Vega et al., 2016). To date, various techniques have been proposed for GSH detection, such as fluorescence spectroscopy (Liu et al., 2016; Dong et al., 2017), high-performance liquid chromatography (HPLC) (Giustarini et al., 2004; Patterson et al., 2008), mass spectrometry (Huang and Chang, 2007; Zheng et al., 2007), absorbance spectroscopy (Li et al., 2010; Liu et al., 2013) and capillary electrophoresis (Musenga et al., 2007). All of these methods, absorbance spectroscopy has attracted more and more attention owing to its simplicity, lowcost, and convenience. In addition, nanomaterials as mimetic peroxidases have become a focus area for research, including V_2_O_5_ nanowires (André et al., 2011), 3D porous graphene nanocomposites (Wang et al., 2017) and Fe_3_O_4_ NPs (Liang and Yan, 2019) etc. The mimetic peroxidase can oxidize the substrate 3,3′,5,5′-tetramethyl benzidine (TMB) in the presence of hydrogen peroxide (H_2_O_2_) with a colorimetric change from colorless to blue, which can be observed by the naked eye and analyzed using ultraviolet-visible spectrophotometry (UV-vis) spectrophotometry. Nanomaterials are expected to perform multi-enzyme functions to achieve multiple uses of an enzyme, thus improving catalytic efficiency (Dong et al., 2014; Fan et al., 2018), or to achieve cascade catalysis, which often has greater advantages and application prospects. Currently, some nanomaterials have been reported to exhibit multi-enzyme-mimicking activity, such as Co_3_O_4_ nanoplates (Wang H. et al., 2018), NiPd hNPs (Wang et al., 2016) and Co_1.5_Mn_1.5_O_4_ (Liu et al., 2021), etc. They can mimic either two, three, or all of the following four redox enzymes: peroxidase, oxidase, catalase, and superoxide dismutase. In addition to these enzymes, the less investigated simulated nanozymes include V_2_O_5_ with peroxidase-like and glucose oxidase (Gox)-like catalytic properties (Ding et al., 2020) and Cu_2_O NPs with cytochrome *c* oxidase activity (Chen M. et al., 2017). Therefore, the development of new nanomaterials with multi-functional enzyme-mimicking properties is necessary.

As biocompatible carbon-based materials, nanodiamonds (NDs) have unique intrinsic properties such as superior hardness and chemical inertness (Grichko et al., 2008; Aleksenskiy et al., 2010; Shenderova et al., 2011). Owing to small size and facile surface functionalization, NDs exhibit lower cytotoxicity and superior biocompatibility than those exhibited by other carbon materials (Xing and Dai, 2009; Zhang et al., 2012; Qin et al., 2021). NDs and their derivatives have recently become interesting topics for cutting-edge research and revealed high application potential in biomedical fields, such as bioimaging, biosensing, implant coating, and drug delivery (Shimkunas et al., 2009; Narayan et al., 2011; Haziza et al., 2017; Su et al., 2019; Fang et al., 2020; Jariwala et al., 2020; Nowicki and Czarniewska, 2020). In addition to the bio-related applications, NDs with reactive oxygen-containing surfaces have exhibited a certain level of antibacterial effects (Wehling et al., 2014; Ong et al., 2018). Meanwhile, oxygenated NDs also emerged as multi-enzyme mimics under various reaction conditions (Chen T. M. et al., 2017; Fang et al., 2020). Furthermore, it was reported that oxygen-containing groups of NDs including carbonyl, carboxyl and hydroxyl groups are the active sites for the release of hydroxyl radical from H_2_O_2_ during oxidative dehydrogenation reaction (Sun et al., 2015; Wang Q. et al., 2018).

**SCHEME 1:**
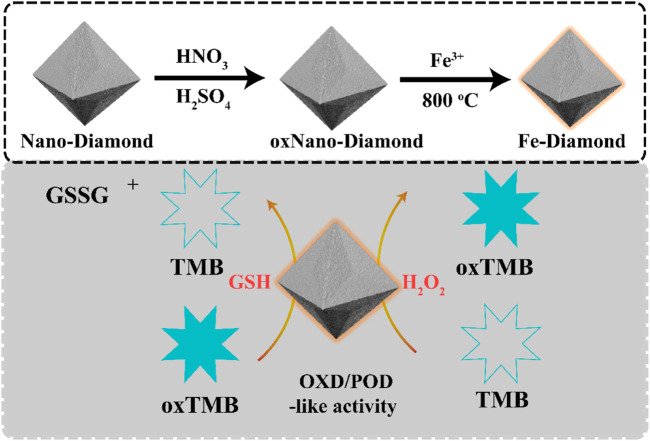
Schematic presentation for Fe-NDs with peroxidase-like and oxidase-like activity.

In the present study, we successfully prepared a single-atom Fe-modified NDs *via* a one-pot insitu reduction method. The effects of single-atom Fe on the visible optical absorption and charge carrier separation as well as Fe-NDs with peroxidase-like and oxidase-like activities have not yet been reported. In this study, the corresponding properties of Fe-NDs were investigated in detail. Moreover, in environments of harsh pH and high temperature, Fe-NDs exhibit excellent catalytic capability and stability, enabling them very suitable for practical application. In the presence of H_2_O_2_, Fe-NDs catalyzed the reaction of TMB to generate colored oxidation products, which were used for detecting H_2_O_2_. In the absence of H_2_O_2_, Fe-NDs oxidized colorless TMB to blue-colored oxidized TMB (oxTMB), which was reduced to colorless TMB by adding different concentrations of GSH (Scheme 1). The whole process can be observed with the naked eye and analyzed using UV-vis spectroscopy. Importantly, the process exhibits a good linear relationship in the concentration range of 1–25 μM with a detection limit of 0.072 μM. Our sensor successfully determined reduced GSH in tablet and injection samples, and the results were also confirmed by using HPLC-UV.

## Experimental

### Materials

Nano-diamond, FeCl_3_, H_2_SO_4_ and HNO_3_, 3,3′,5,5′-tetramethylbenzidine (TMB), 1,2-diaminobenzene (OPD), 2,2′-azinobis-(3-ethylbenzthiazoline-6-sulphonate) (ABTS), L-γ-glutamyl-L-cysteinylglycine (GSH), thiourea were purchased from Macklin reagent Co.,Ltd. (Shanghai, China), Human serum albumin (HAS), Bovine serum albumin (BSA), ascorbic acid (AA), glycine (Gly), L-lysine (Lys), L-serine (Ser), D/L-cysteine(D-Cys, L-Cys), glucose, sodium chloride, calcium chloride, cupric sulfate, ferric chloride, potassium chloride, zinc sulfate, magnesium sulfate, tartaric acid, choline chloride were purchased from Sinopharm Chemical Reagent Co. Ltd. NaN_3_ was purchased fromTianjin Fuchen Chemical Reagent Factory. H_2_O_2_ was purchased from Xilong Scientific Co., Ltd. sodium chloride, calcium chloride, cupric sulfate, ferric chloride, potassium chloride, zinc sulfate, magnesium sulfate, tartaric acid, choline chloride was purchased fromSinopharm ChemicalReagent Co., Ltd. Sodium acetate buffer (0.1 M, pH = 4.0) were freshly prepared before use. All solutions prepared for purified water are derived from Wahaha purified water (China).

### Instrumentation

X-ray powder diffraction (XRD) was collected on a PANalytical B.V. Empyrean powder diffractometer, in which data were collected from 5° to 80° at a scan rate of 10°/min. Scanning electron microscopy (SEM) images were captured on a Hitachi FE-SEM S-4800 instrument with an acceleration voltage of 3 kV. Transmission electron microscopy (TEM) and high-resolution TEM (HRTEM) images was carried on a JEM-2100F. Spherical aberration corrected Transmission Electron Microscope (ACTEM) was carried on a JEM-ARM300F. UV-vis spectra were measured on UV-2700 Spectrophotometer (Shimadzu, Japan).

### Synthesis of Fe-NDs

The purchased NDs (0.2 g) were dispersed in a mixture (20 ml) of H_2_SO_4_ and HNO_3_ with a volume ratio of 3:1 and heated to 90°C for 2 h. After the heating, the suspensions were cooled down to room temperature and neutralized by the adding of NaOH solution. After dialysis against water, the above suspensions were dispersed in HCl solution with a final concentration of 0.1 M and heated at 90°C for another 2 h. O-NDs with abundant specific oxygenated groups were obtained after proper dialysis and freeze-drying. Fe in the as-prepared O-NDs were added into 20 ml distilled water. The obtained solution was heated at 90°C for 1 h under stirring, then the temperature was raised to 100°C for complete water evaporation. The resulting mixture was put into an alumina crucible with a cover, and heated to 700°C with the ramping rate of 20°C/min, and kept at that temperature for another 4 h. This process was conducted with 30 ml/min N_2_ flow at atmospheric pressure. Nano-diamond modified with single-atom Fe is denoted as Fe-NDs.

### Enzyme Mimicking Activities of Fe-NDs

Fe-NDs with peroxidase-like activity can directly oxidize substrates in the presence of H_2_O_2_. The whole reaction system consists of 44 μl of 0.18 mg ml^−1^ Fe-NDs, 50 μl H_2_O_2_ (1 mM) and 100 μl of 4 mM, TMB were added to 806 μl of 0.1 M HAC-NaAC buffer solution (pH = 4.0). Finally, the mixed system was reacted at 55°C for 15 min and the UV absorption was measured at 652 nm.

The detective process of oxidase-like activity is similar to that of peroxidase mimics, except that no H_2_O_2_ is added. To assess the oxidase activity of Fe-NDs, Typically, 100 μl of 0.18 mg ml^−1^ Fe-NDs and 100 μl of 4 mM TMB were added to 800 μl of 0.1 M HAC-NaAC buffer solution (pH = 4.0). Finally, the mixed system was reacted at 40°C for 20 min and the UV absorption was measured at 652 nm.

### Steady-State Kinetic Analysis

The steady-state kinetics experiment of peroxide-like properties was carried out with Fe-NDs suspension (44 μl, 0.18 mg ml^−1^), H_2_O_2_ (50 μl, 1 mM), and TMB (100 μl, 4 mM). The mixed system was reacted at 55°C for 10 min before being used directly for UV-vis absorbance measurements. Similarly, kinetic analysis of the oxidase-like properties was carried out with Fe-NDs suspension (100 μl, 0.18 mg ml^−1^) by varying the concentration of TMB. The mixed system was reacted at 40°C for 10 min before being used directly for UV-vis absorbance measurements.

A typical experimental operation is to determine the reaction rate changes with different concentrations of TMB under optimal conditions. The kinetic parameters are determined by the following equations: 1/ν = *K*_m_/*V*_max_⋅(1/[S] + 1/*K*_m_), where ν is the initial velocity, *V*_max_ is the maximal reaction velocity, and [S] is the concentration of the substrate. *K*_m_ is the Michaelis–Menten constant, which indicates the enzyme affinity for the substrate.

### Colorimetric Detection of Hydrogen Peroxide and Glutathione

The working solution for H_2_O_2_ determination as follows: 44 μl Fe-NDs suspension (0.18 mg ml^−1^), 100 μl TMB (4 mM) and different concentrations of H_2_O_2_ (1–60 μM, 50 μl) were added into 806 μl of 0.1 M HAc-NaAc buffer solution (pH = 4.0). Then, the absorbance of the mixed solution at 652 nm was measured after incubation for 15 min at 55°C temperature.

The whole reaction system for GSH determination consisted of 100 μl TMB (4 mM), 500 μl of 0.1 M HAc-NaAc buffer solution (pH = 4.0) and 100 μl of Fe-NDs suspension (0.18 mg ml^−1^). After 20 min of reaction at 40°C temperature, the 300 μl GSH solution was added, and then the absorbance is recorded on UV-vis spectra at 625 nm after 15 min. GSH concentration is calculated by measuring the change in absorbance (ΔA) of the reaction system after adding GSH. The blank group was given the same amount of ultrapure water instead of GSH.

### Detection of GSH in Drug Samples

The glutathione tablets and injections were produced by Chongqing Yaoyou Pharmaceutical Co., LTD. The tablets and injections were prepared with a certain concentration of GSH test solution, followed by the addition of different concentrations of GSH standard solution to 5, 10, and 15 μM, so that the concentrations were detected in a linear range. The detection method of GSH was performed in accordance with 2.6*.*

## Results and Discussion

### Construction and Characterization of Fe-NDs

**FIGURE 1:**
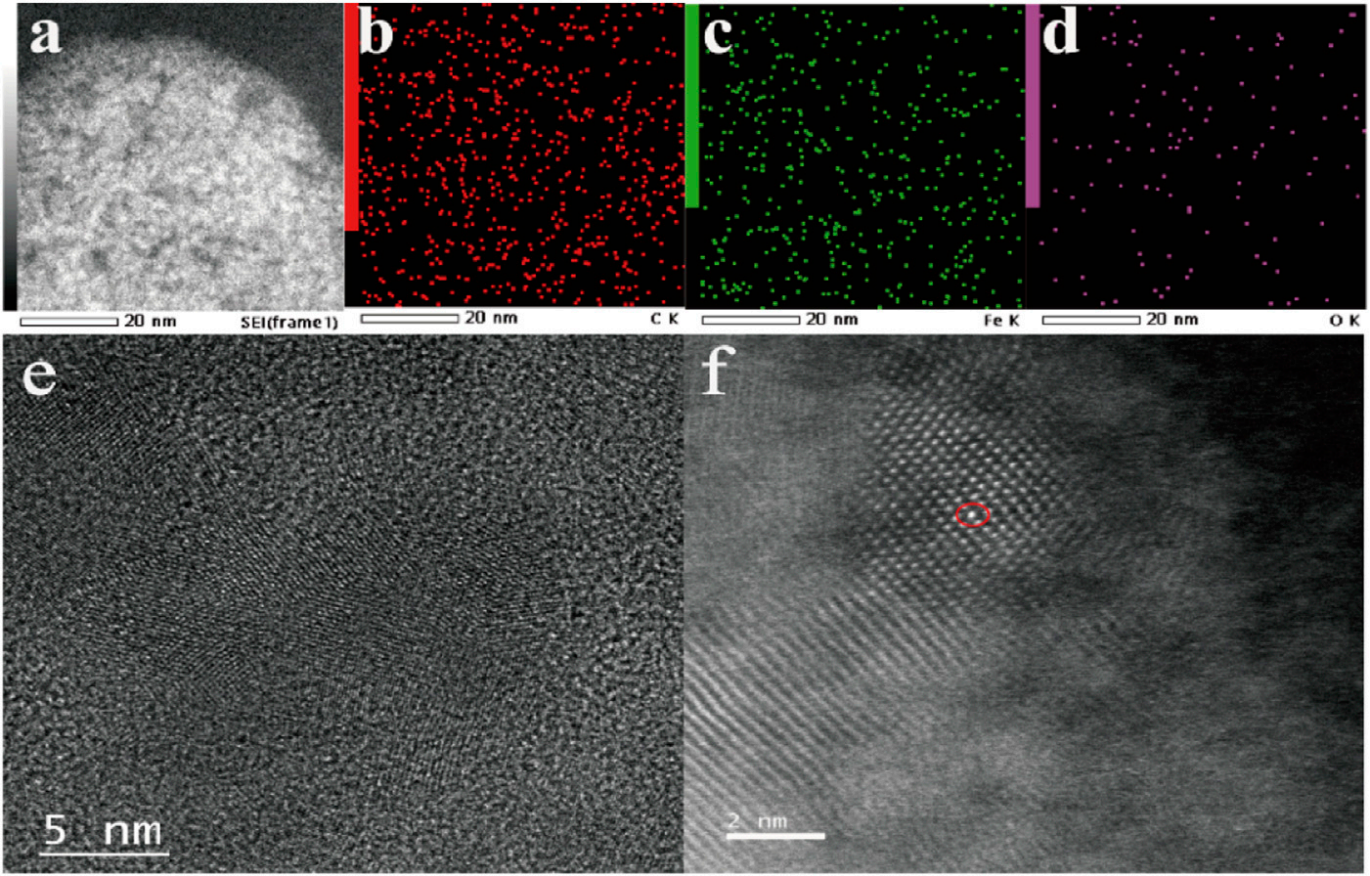
**(A)** TEM images of Fe-ND; **(B)** Corresponding elemental mappings of component elements C, **(C)** Fe, **(D)** O; **(E)** HRTEM image of Fe-ND; **(F)** ACTEM of image of Fe-ND.

Typically, Fe-NDs were well synthesized with the oxidized NDs asprecursors *via* coordination with iron, and the composite was further annealing at 800°C. The presence of diamond in the sample is confirmed by the XRD pattern Supplementary Figure S1 (JCPDS No. 75-0219) (Chen T. M. et al., 2017). The size of the synthesized nanoparticles were irregular lamellar structure (Supplementary Figure S2). As demonstrated in Figures 1A–D, C, O, and Fe elements coexisted on the surface of NDs. These results suggest that Fe may exist as a single atom. In order to further verify the existence of Fe single atom, Fe atoms in Fe-NDs samples were directly observed by using spherical aberration corrected Transmission Electron Microscope (ACTEM). As shown in Figures 1E,F, oxidized nano-diamond has lattice structure and abundant single Fe atoms are clearly observed as bright dots, indicating that Fe single atoms were successfully single dispersed on NDs.

### Peroxidase-Like Activity of Fe-NDs

**FIGURE 2:**
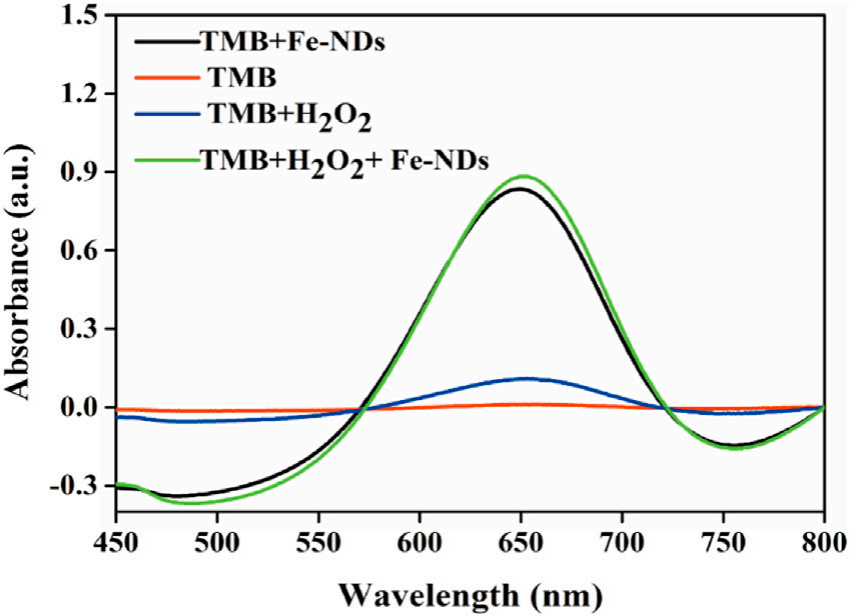
Typical absorption spectra in different reaction systems of TMB, TMB + H_2_O_2_, TMB + Fe-NDs, and TMB + H_2_O_2_ + Fe-NDs.

Several typical substrates {i.e., TMB, OPD (1,2-diaminobenzene), and ABTS [2,2′-azinobis-(3-ethylbenzthiazoline-6-sulphonate)]} were used to investigate the peroxidase activity of the synthesized Fe-NDs. Supplementary Figure S3 demonstrates that in the presence of H_2_O_2_, the three colorless substrates (TMB, OPD, and ABTS) were oxidized and turned blue, yellow, and green, respectively, indicating the presence of peroxidase activity in Fe-NDs. TMB was selected as the substrate to further confirm the results. Figure 2 demonstrates that in the absence of H_2_O_2_, TMB alone could not form chromogenic products. However, when Fe-NDs, TMB, and H_2_O_2_ were present in the reaction system, an increasing absorption was observedat 625 nm, which indicated that Fe-NDs acted as peroxidase-like mimics in the catalytic reaction between TMB and H_2_O_2_.

**FIGURE 3:**
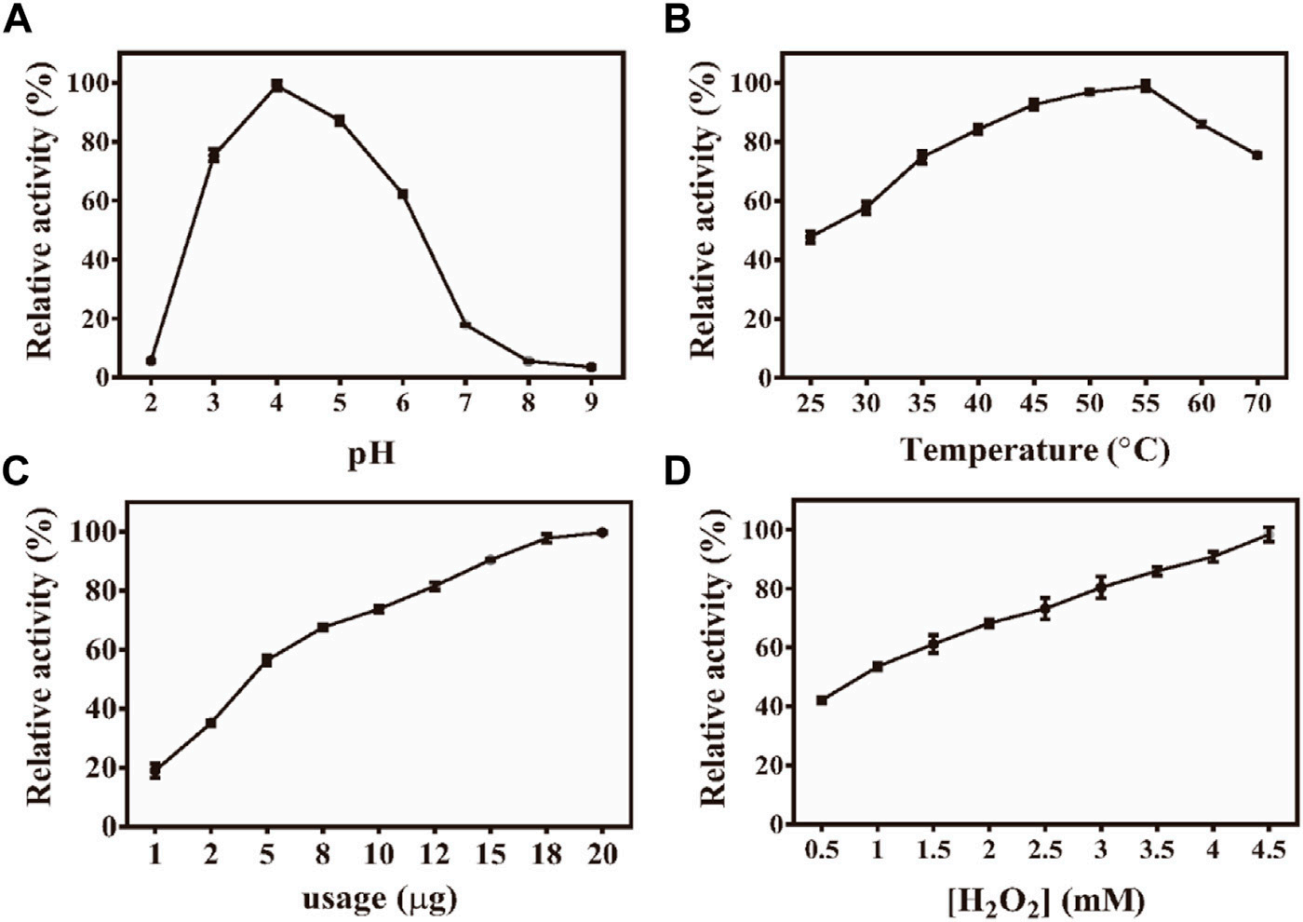
**(A)** Effect of pH on the activity of Fe-NDs + H_2_O_2_ + TMB system; **(B)** Effect of temperature on the activity of Fe-NDs + H_2_O_2_ + TMB system; **(C)** Effect of catalyst dosage on the activity of Fe-NDs + H_2_O_2_ + TMB system; **(D)** Effect of H_2_O_2_ concentrationon the activity of Fe-NDs + H_2_O_2_ + TMB system. The error bars are the SD of the third parallel sample.

Similar to the properties of horseradish peroxidase (HRP), the peroxidase-like properties of Fe-NDs depend on pH, temperature, and H_2_O_2_ concentration. The activity of the material was measured at a pH of 2–9 and a temperature of 25–70°C (Figures 3A–D). Figure 3A demonstrates that the catalytic activity of Fe-NDs was greatly affected by pH, and the highest activity was at a pH of 4, which is similar to the activity of HRP and other reported peroxide-like enzymes (Zhang et al., 2013; Xia et al., 2015; Wang et al., 2017). In addition, Fe-NDs maintained a high catalytic activity in a wide range of temperatures. As demonstrated in Figure 3B, Fe-NDs maintained more than 80% catalytic activity in the temperature range of 35–60°C. The catalytic activity of Fe-NDs increased with an increase in the amount of material and concentration of H_2_O_2_ (Figure 3D). Eventually, the optimal experimental conditions were determined to be a pH of 4.0, a temperature of 55°C, and a material concentration of 8 μg ml^−1^.

### Oxidase-Like Activity of Fe-NDs

Oxidases oxidize the peroxidase substrate TMB to produce blue-colored oxTMB. Oxidase activity was found in studying the peroxide-like activity of Fe-NDs, which was confirmed by TMB turned blue directly in the absence of H_2_O_2_ (Figure 2). To study the oxidase activity of NDs before and after the addition of Fe, the change in absorbance at 652 nm was monitored using a UV-vis spectrometer. As demonstrated in Supplementary Figure S4, Fe-NDs significantly catalyzed TMB to produce a blue-colored reaction without H_2_O_2_. TMB is oxidized to oxTMB by oxygen in the presence of Fe-NDs, with a concomitant visible colorimetric change that can be observed by the naked eye. However, untreated NDs did not exhibit oxidase activity, indicating that the mixed acid oxidation process of NDs and the introduction of Fe played a key role in the oxidase activity of NDs. Fe-NDs can also oxidise different color-developing substrates (ABTS, which turns green, and OPD, which turns yellow) under certain conditions without adding H_2_O_2_, as demonstrated in Supplementary Figure S5. Therefore, the results indicated that Fe-NDs exhibited significant oxidase-like catalytic activity and directly catalysed the substrate.

**FIGURE 4:**
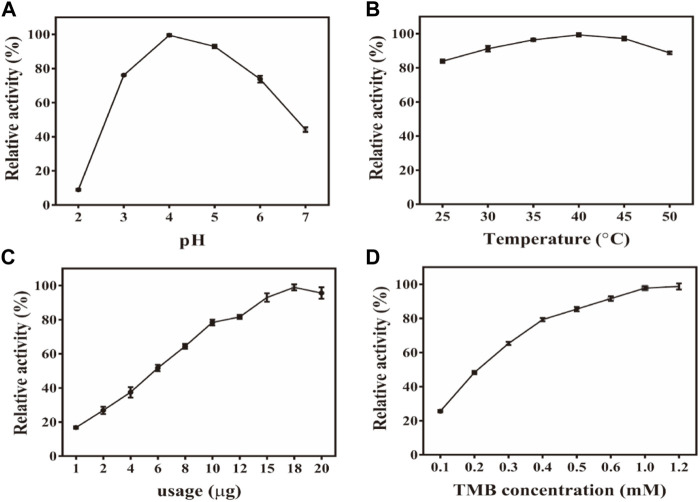
**(A)** Effect of pH on the activity of Fe-NDs -TMB system; **(B)** Effect of temperature on the activity of Fe-NDs -TMB system; **(C)** Effect of catalyst dosage on the activity of Fe-NDs -TMB system; **(D)** Effect of TMB concentration on the activity of Fe-NDs -TMB system. The error bars are the SD of the third parallel sample.

Subsequently, the effects of different reaction conditions on the oxidase activity of Fe-NDs were studied. Parameters such as catalyst concentration, pH, temperature, and TMB concentration were investigated (Figures 4A–D). To analyze the influence of pH on the catalytic activity of Fe-NDs, pH ranging from 2 to 7 was used in the colorimetric experiment. The results revealed that the optimal pH for the catalytic activity of Fe-NDs was 4.0. The temperature range of 25–60°C was used to analyze the influence of temperature on the catalytic activity of Fe-NDs. As demonstrated in Figure 4B, the catalytic activity of Fe-NDs remained above 80%, proving that Fe-NDs exhibited catalytic activity over a wide temperature range. Eventually, 40°C was selected as the optimal temperature for follow-up experiments. In addition, we analyzed the influence of catalyst concentration ranging from 1 to 20 μg ml^−1^ on the activity of Fe-NDs. The results revealed that the catalytic activity of Fe-NDs increased rapidly when the concentration was 1–10 μg ml^−1^, and the growth rate was slow when the concentration was greater than 10 μg ml^−1^ until the activity reached the highest at 18 μg ml^−1^. Therefore, 18 μg ml^−1^ was determined as the optimal catalyst concentration. At the same time, the catalytic activity of Fe-NDs remained above 80% when the concentration of TMB was higher than 0.4 mM, so 0.4 mM was selected as the optimal substrate concentration. The optimal experimental conditions were as follows: temperature, 40°C; pH, 4.0; material concentration, 18 μg ml^−1^ and TMB concentration, 0.4 mM.

### Kinetic Analysis of Fe-NDs and Exploration of Reactive Oxygen Species

**FIGURE 5:**
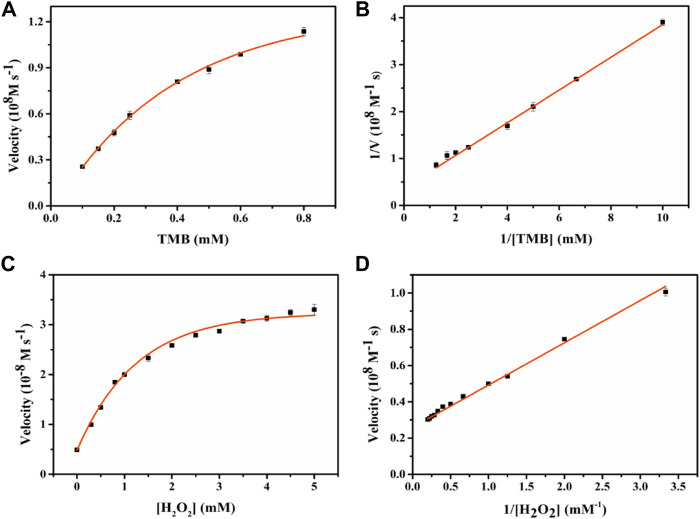
Enzyme kinetics of Fe-NDs for POD-like activity. The concentration of H_2_O_2_ in **(A)** was 1 mM and the TMB in **(C)** was 0.4 mM. **(A)** Kinetic plot of ν against TMB concentration; **(B)** Double reciprocal plot from **(A)**; **(C)** Kinetic plot of ν against H_2_O_2_ concentration; **(D)** Double reciprocal plot from **(C)**.The error bars are the standard deviation of the third parallel sample.

To evaluate the peroxidase-like catalytic performance of Fe-NDs, the steady-state kinetic parameters were analyzed by changing the concentration of TMB and H_2_O_2_ in the reaction system. The absorbance of the TMB oxidation product, ε = 39 000 M^−1^ cm^−1^ (652 nm), was used to calculate the concentration of the substance corresponding to the absorbance. A typical Michaelis-Menten curve is shown in Figure 5, and the maximum initial velocity (*V*_max_) and Michaelis–Menten constant (*K*_m_) are provided in Supplementary Table S1. It was observed that Fe-NDs exhibited a stronger affinity in terms of H_2_O_2_ (*K*_m_ = 0.87 mM) compared to horseradish peroxidase (HRP) (*K*_m_ = 3.7 mM). Furthermore, the *K*_m_ value of Fe-NDs was 0.76 mM when TMB was used as the substrate, suggesting that a higher concentration of TMB is required to achieve maximal enzymatic activity for the prepared nanozyme.

To better understand the catalytic mechanism of POD-like nanozyme, we used some free radical trapping agents. Results as shown in Supplementary Figure S6, p-benzoquinone, NaN_3_, and thiourea were captured by the superoxide radicals (O_2_·^−^), singlet oxygen molecules (^1^O_2_), and hydroxyl free radicals (OH·), respectively. In the presence of thiourea in the system, the catalytic activity of Fe-NDs is significantly lower than that of the blank. The addition of p-benzoquinone can reduce the catalytic activity by about 10%. The main active substance produced in the TMB oxidation process is OH· and a little O_2_^−^· is also produced in this process.

**FIGURE 6:**
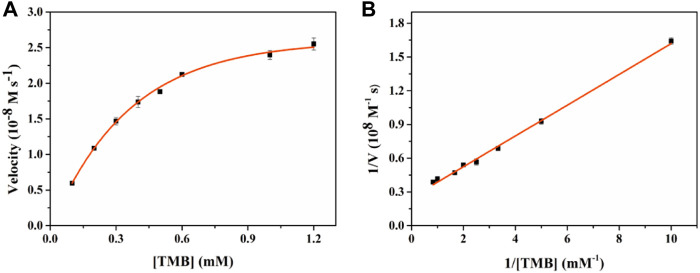
Enzyme kinetics of Fe-NDs for OXD-like activity. The concentration of H_2_O_2_ in **(A)** was 1 mM. **(A)** Kinetic plot of ν against TMB concentration; **(B)** Double reciprocal plot from **(A)**. The error bars are the standard deviation of the third parallel sample.

To further evaluate the oxidase-like catalytic performance of Fe-NDs, the kinetic experiment was performed by changing the concentration of TMB under optimal experimental conditions. A typical Michaelis–Menten curve is demonstrated in Figure 6, and the maximum initial velocity *V*_max_ and *K*_m_ calculated are provided in Supplementary Table S2. The values of *K*_m_ and *V*_max_ were 0.55 mM and 4.01 × 10^−8^ M s^−1^, respectively, when TMB was used as the substrate. Compared with the classic CeO_2_ NPs, Fe-NDs had a higher affinity for TMB, which may be attributed to the uniform dispersion of Fe atoms on the diamond surface enhancing its catalytic properties.

Oxygen plays an important role as an electron acceptor in the catalytic activity of oxidases. The reaction solution was pre-treated with nitrogen and oxygen for half an hour to confirm the role of oxygen in catalysis, and Fe-NDs were subsequently added to catalyze TMB under optimal conditions. As demonstrated in Supplementary Figure S7, under the saturation condition of nitrogen, the catalytic activity of Fe-NDs was significantly inhibited and was only 40%. However, under the saturation condition of oxygen, the catalytic activity was significantly increased to 72% compared with that in the air, which proved that oxygen played an important role in the oxidation of TMB. The oxidase activity of Fe-NDs may be attributed to the reactive oxygen species (ROS) produced during the oxidation of TMB. To analyze the influence of different free radicals on the reaction system, we used different concentrations of p-benzoquinone, NaN_3,_ and thiourea to scavenge thesuperoxide radicals (O_2_·^−^), singlet oxygen molecules (^1^O_2_), and hydroxyl free radicals (OH·), respectively. The results are demonstrated in Supplementary Figure S8, the three different concentrations of trapping agents can inhibit the catalytic activity of Fe-NDs, indicating that the system produces three kinds of ROS, which are O_2_·^−^, ^1^O_2,_ and OH· respectively.

### Colourimetric Assessment of H_2_O_2_

**FIGURE 7:**
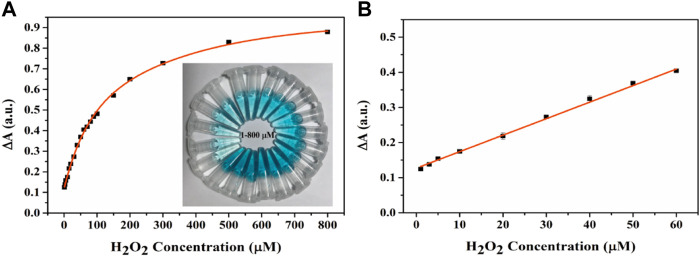
**(A)** The UV-vis spectra and corresponding color changes (inset image) of the Fe-NDs + TMB system in the presence of a various concentrations of H_2_O_2_. **(B)** Good linear calibration plots for H_2_O_2_ detection. The error bars are the SD of the third parallel sample.

H_2_O_2_ has been associated with cell damage and several diseases (Song et al., 2010; Fu et al., 2014). Therefore, it is important to establish a simple, highly sensitive, rapid technique for the visual detection of H_2_O_2_. The experimental results of H_2_O_2_ detection by Fe-NDs colorimetric method are demonstrated in Figures 7A,B, it demonstrated that the absorbance of TMB increases with an increase in H_2_O_2_ concentration and exhibits a good linear relationship with H_2_O_2_ concentration (1–60 μM; R^2^ = 0.9989). According to the 3σ rule the detection limit for H_2_O_2_ was calculated to be 0.3 μM, indicating that the H_2_O_2_ sensor had a low detection limit. As demonstrated in Figure 7A, the color of the solution deepened with increasing H_2_O_2_ concentration, indicating that the sensor exhibited excellent visual detection performance. Compared with other sensors based on peroxide-like activity used for detecting H_2_O_2_, as mentioned in Supplementary Table S3, the colorimetric method used in this study has a higher sensitivity and lower detection limit.

### Colourimetric Assessment of GSH

GSH is a typical reducing agent that can directly reduce blue-colored oxTMB to colorless TMB owing to its rich mercapto functional groups (Liu et al., 2013). Differences in absorbance (ΔA) before and after adding GSH exhibited a good linear relationship with the concentration of GSH in the solution within a certain range. Based on changes in the absorbance value before and after (ΔA) detection, the Fe-NDs colorimetric biosensor was established to quantitatively detect GSH. To determine the optimal conditions for GSH detection, we studied the effects of pH, temperature, and material concentration on the catalytic activity of Fe-NDs in the presence of GSH (20 μM) (Supplementary Figure S9). As demonstrated in Supplementary Figure S9A, pH had an impact on the reaction system. The highest relative activity was achieved by deducting blank when pH was 4.0. Therefore, a pH of 4.0 was considered optimal for subsequent experiments. No significant difference was observed in the value of ΔA in the temperature range of 25–50°C (Supplementary Figure S9B). Considering the optimal detection conditions, 40°C was selected as the optimal temperature. Based on the combined results of detection using different pH, temperature, catalyst dosage, and TMB concentration (Supplementary Figure S9D), the optimal conditions for GSH detection were as follows: pH, 4.0; temperature, 40°C; Fe-ND concentration, 18 μg ml^−1^ and TMB concentration, 0.4 mM.

**FIGURE 8:**
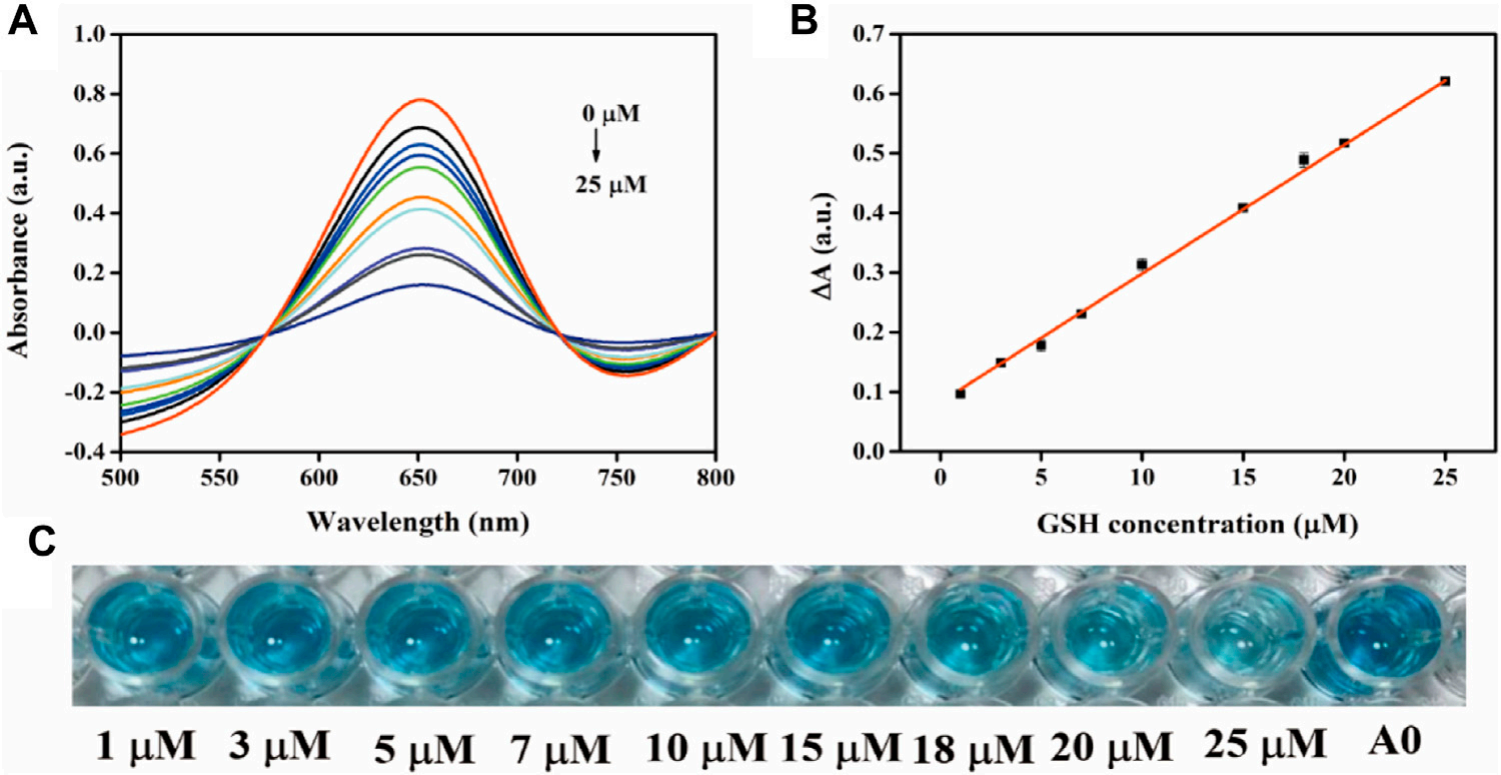
**(A)** UV-vis spectra of the sensing system with different GSH concentrations; **(B)** Linear plots of ΔA versus GSH concentration; **(C)** an overview photograph.

A simple, fast, and sensitive visual colorimetric sensor for GSH detection can be established based on the properties of oxidases. Figure 8A demonstrates the UV-vis spectrum curve for detecting the absorption value of GSH in the concentration range of 1–25 μM at 652 nm. The corresponding calibration curve in the range of 1–25 μM (R^2^ = 0.9997) is demonstrated in Figure 8B. A good linear relationship was observed between ΔA and GSH concentration, and the equation was as follows: ΔA = 0.01 [GSH] (μM) + 0.027. The detection limit for GSH was 0.072 μM according to the three-sigma (3σ) rule, which indicates that the colorimetric sensor can be used for reliable detection of GSH in real samples. As demonstrated in Figure 8C, the color of the solution gradually became lighter as the concentration of GSH increased, indicating that the modified sensor had adequate visual detection performance. The Fe-ND-based sensor had a lower detection limit than the previously published nanoenzyme-based colorimetric GSH sensors mentioned in Supplementary Table S4, indicating the high sensitivity of the method.

### Selectivity and Stability of Fe-NDs

**FIGURE 9:**
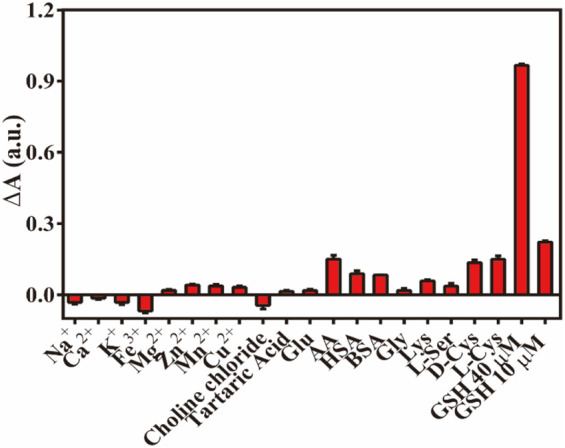
The ΔA responses of Fe-NDs + TMB system towards GSH and interferents (HAS and BSA, 1 mg ml^−1^; others,1 mM). Error bar represents the standard deviation for three determinations.

To evaluate the anti-interference performance of the established colorimetric sensor, various co-existing disturbance species were tested under the same conditions, including various metal ions (Na^+^, K^+^, Ca^2+^, Fe^3+^, Mg^2+^, Cu^2+^, Zn^2+^, and Mn^2+^), amino acids [glycine, lysine, L-serine and D/L(+)-cysteine], HAS, BSA, glucose, tartaric acid, choline chloride, and ascorbic acid. As demonstrated in Figure 9, the concentration of these cationic interfering species and amino acids was 100 times (1 mM) that of GSH, and their influence on the absorbance value of the Fe-NDs/TMB system was negligible. Some biological macromolecules such as BSA (1 mg/mL) and HSA (1 mg/mL) had also little influence on the system. The absorbance values (ΔA) of AA and D/L(+)-cysteine were similar to those of the 10 μM GSH solution; however, the difference in concentration of AA (1 mM) and D/L(+)-cysteine (1 mM) and GSH (10 μM) solution was 100 times, and its effect could be ignored. Therefore, the proposed method has a higher selectivity for GSH detection and can be widely used for the rapid quantification of biological and biomedicine samples.

As demonstrated in Supplementary Figure S10, the stability of the oxidase-like activity of Fe-NDs was investigated. The catalytic activity of Fe-NDs remained above 80% at room temperature for 60 days, indicating that the material has good stability.

### Application of the GSH Sensor

We used some drug samples to demonstrate the feasibility of this method to detect GSH in a complex environment and the results are provided in Supplementary Table S5. Statistical analysis indicate that the recovery rates of GSH in tablets and injections were in the range of 94.6–101.5% (RSD, 1.5–1.9%) and 97.2–98.5% (RSD, 1.2–2.0%), respectively (Table 1). All these results demonstrate that the proposed method is reliable for practical applications. At the same time, HPLC-UV was used to verify the accuracy of the Fe-NDs colorimetric sensor (National Pharmacopoeia Committee, 2020). The final results showed that the Fe-NDs colorimetric sensor designed in this paper has good accuracy and sensitivity which can be used to detect GSH in actual samples.

**TABLE 1:** Results of GSH analysis for tablets and injection solutions.

Sample	Found in sample (μM)	Spiked (μM)	Found (μM)	Recovery (%)	RSD (%)
Tablets	5.09	—	—	—	—
—	—	5.00	10.16	101.5	1.9
—	—	10.00	15.07	99.8	1.7
—	—	15.00	19.27	94.6	1.5
Injection	4.96	—	—	—	—
—	—	5.00	9.88	98.4	2.0
—	—	10.00	14.81	98.5	1.2
—	—	15.00	19.54	97.2	1.8

## Conclusion

In conclusion, this study is the first of its kind to demonstrate the preparation of two-dimensional lamellar nanostructures containing iron using a hydrothermal method. The method is simple and environment-friendly. The prepared Fe-NDs could mimic two types of enzymes with peroxidase-like and oxidase-like activities. Studies have demonstrated that Fe-NDs exhibit excellent catalytic activity and long-term stability in harsh environments. Improvement in the catalytic activity of Fe-NDs is mainly attributed to the introduction of Fe. Based on the enhanced catalytic activity, we successfully constructed a novel H_2_O_2_ sensor and GSH detector. The proposed Fe-NDs nanozyme-based visual sensing platform exhibits satisfying sensitivity, selectivity, and stability. This study provides a novel method for the preparation of various nanozyme materials and promotes the development and application of nanozymes in chemical and medical diagnosis.

## Data Availability

The original contributions presented in the study are included in the article/Supplementary Material, further inquiries can be directed to the corresponding author.

## References

[B1] Aleksenskiy A. Baidakova M. Osipov V. Vul’ A. (2010). The Fundamental Properties and Characteristics of Nanodiamonds. Nanodiamonds 55, 77. 10.1007/978-1-4419-0531-4_3

[B2] André R. Natálio F. Humanes M. Leppin J. Heinze K. Wever R. (2011). V2O5 Nanowires with an Intrinsic Peroxidase-like Activity. Adv. Funct. Mater. 21, 501–509. 10.1002/adfm.201001302

[B3] Chen M. Wang Z. Shu J. Jiang X. Wang W. Shi Z.-H. (2017a). Mimicking a Natural Enzyme System: Cytochrome C Oxidase-like Activity of Cu2O Nanoparticles by Receiving Electrons from Cytochrome C. Inorg. Chem. 56, 9400–9403. 10.1021/acs.inorgchem.7b01393 28753305

[B4] Chen T. M. Tian X. M. Huang L. Xiao J. Yang G. W. (2017b). Nanodiamonds as pH-Switchable Oxidation and Reduction Catalysts with Enzyme-like Activities for Immunoassay and Antioxidant Applications. Nanoscale 9, 15673–15684. 10.1039/c7nr05629j 28994431

[B5] Ding Y. Ren G. Wang G. Lu M. Liu J. Li K. (2020). V2O5 Nanobelts Mimick Tandem Enzymes to Achieve Nonenzymatic Online Monitoring of Glucose in Living Rat Brain. Anal. Chem. 92, 4583–4591. 10.1021/acs.analchem.9b05872 32056429

[B6] Dong J. Song L. Yin J.-J. He W. Wu Y. Gu N. (2014). Co3O4 Nanoparticles with Multi-Enzyme Activities and Their Application in Immunohistochemical Assay. ACS Appl. Mater. Inter. 6, 1959–1970. 10.1021/am405009f 24387092

[B7] Dong Z.-Z. Lu L. Ko C.-N. Yang C. Li S. Lee M.-Y. (2017). A MnO2nanosheet-Assisted GSH Detection Platform Using an Iridium(iii) Complex as a Switch-On Luminescent Probe. Nanoscale 9, 4677–4682. 10.1039/C6NR08357A 28139807

[B8] Fan K. Xi J. Fan L. Wang P. Zhu C. Tang Y. (2018). *In Vivo* guiding Nitrogen-Doped Carbon Nanozyme for Tumor Catalytic Therapy. Nat. Commun. 9, 1440–1451. 10.1038/s41467-018-03903-8 29650959 PMC5897348

[B9] Fang J. Wang H. Bao X. Ni Y. Teng Y. Liu J. (2020). Nanodiamond as Efficient Peroxidase Mimic against Periodontal Bacterial Infection. Carbon 169, 370–381. 10.1016/j.carbon.2020.07.055

[B10] Fu P. P. Xia Q. Hwang H.-M. Ray P. C. Yu H. (2014). Mechanisms of Nanotoxicity: Generation of Reactive Oxygen Species. J. Food Drug Anal. 22, 64–75. 10.1016/j.jfda.2014.01.005 24673904 PMC9359151

[B11] Giustarini D. Dalle-Donne I. Colombo R. Milzani A. Rossi R. (2003). An Improved HPLC Measurement for GSH and GSSG in Human Blood. Free Radic. Biol. Med. 35, 1365–1372. 10.1016/j.freeradbiomed.2003.08.013 14642384

[B12] González de Vega R. Fernández-Sánchez M. L. Fernández J. C. Álvarez Menéndez F. V. Sanz-Medel A. (2016). Selenium Levels and Glutathione Peroxidase Activity in the Plasma of Patients with Type II Diabetes Mellitus. J. Trace Elem. Med. Biol. 37, 44–49. 10.1016/j.jtemb.2016.06.007 27473831

[B13] Grichko V. Tyler T. Grishko V. I. Shenderova O. (2008). Nanodiamond Particles Forming Photonic Structures. Nanotechnology 19, 225201–225207. 10.1088/0957-4484/19/22/225201 21825753

[B14] Haziza S. Mohan N. Loe-Mie Y. Lepagnol-Bestel A.-M. Massou S. Adam M.-P. (2017). Fluorescent Nanodiamond Tracking Reveals Intraneuronal Transport Abnormalities Induced by Brain-Disease-Related Genetic Risk Factors. Nat. Nanotech 12, 322–328. 10.1038/nnano.2016.260 27893730

[B15] Huang Y.-F. Chang H.-T. (2007). Analysis of Adenosine Triphosphate and Glutathione through Gold Nanoparticles Assisted Laser Desorption/ionization Mass Spectrometry. Anal. Chem. 79, 4852–4859. 10.1021/ac070023x 17523592

[B16] Jariwala D. H. Patel D. Wairkar S. (2020). Surface Functionalization of Nanodiamonds for Biomedical Applications. Mater. Sci. Eng. C 113, 110996. 10.1016/j.msec.2020.110996 32487405

[B17] Jung H. S. Chen X. Kim J. S. Yoon J. (2013). Recent Progress in Luminescent and Colorimetric Chemosensors for Detection of Thiols. Chem. Soc. Rev. 42, 6019–6031. 10.1039/C3CS60024F 23689799

[B18] Li Y. Wu P. Xu H. Zhang H. Zhong X. (2010). Anti-aggregation of Gold Nanoparticle-Based Colorimetric Sensor for Glutathione with Excellent Selectivity and Sensitivity. Analyst 136, 196–200. 10.1039/c0an00452a 20931106

[B19] Liang M. Yan X. (2019). Nanozymes: From New Concepts, Mechanisms, and Standards to Applications. Acc. Chem. Res. 52, 2190–2200. 10.1021/acs.accounts.9b00140 31276379

[B20] Liu T. Huo F. Li J. Chao J. Zhang Y. Yin C. (2016). A Fast Response and High Sensitivity Thiol Fluorescent Probe in Living Cells. Sensors Actuators B: Chem. 232, 619–624. 10.1016/j.snb.2016.04.014

[B21] Liu X. Wang Q. Zhang Y. Zhang L. Su Y. Lv Y. (2013). Colorimetric Detection of Glutathione in Human Blood Serum Based on the Reduction of Oxidized TMB. New J. Chem. 37, 2174–2178. 10.1039/c3nj40897c

[B22] Liu X. Yang J. Cheng J. Xu Y. Chen W. Li Y. (2021). Facile Preparation of Four-In-One Nanozyme Catalytic Platform and the Application in Selective Detection of Catechol and Hydroquinone. Sensors Actuators B: Chem. 337, 129763. 10.1016/j.snb.2021.129763

[B23] Lu S. C. (2009). Regulation of Glutathione Synthesis. Mol. Aspects Med. 30, 42–59. 10.1016/j.mam.2008.05.005 18601945 PMC2704241

[B24] Micke P. Beeh K. M. Schlaak J. F. Buhl R. (2001). Oral Supplementation with Whey Proteins Increases Plasma Glutathione Levels of HIV-Infected Patients. Eur. J. Clin. Invest. 31, 171–178. 10.1046/j.1365-2362.2001.00781.x 11168457

[B25] Musenga A. Mandrioli R. Bonifazi P. Kenndler E. Pompei A. Raggi M. A. (2007). Sensitive and Selective Determination of Glutathione in Probiotic Bacteria by Capillary Electrophoresis-Laser Induced Fluorescence. Anal. Bioanal. Chem. 387, 917–924. 10.1007/s00216-006-0980-6 17203251

[B26] Narayan R. J. Boehm R. D. Sumant A. V. (2011). Medical Applications of diamond Particles & Surfaces. Mater. Today 14, 154–163. 10.1016/s1369-7021(11)70087-6

[B27] National Pharmacopoeia Committee (2020). Pharmacopoeia of People’ S Republic of China, Part 2. Beijing: China Medical Science and Technology Press, 629.

[B28] Nowicki P. Czarniewska E. (2020). Nanodiamenty: Unikalne Nanocząsteczki Do Zastosowania W Biomedycynie I Biotechnologii. Postepy Biochem. 65, 247–262. 10.18388/pb.2019_281 31945279

[B29] Ong S. Y. van Harmelen R. J. J. Norouzi N. Offens F. Venema I. M. Habibi Najafi M. B. (2018). Interaction of Nanodiamonds with Bacteria. Nanoscale 10, 17117–17124. 10.1039/C8NR05183F 30182122

[B30] Patterson A. D. Li H. Eichler G. S. Krausz K. W. Weinstein J. N. Fornace A. J. (2008). UPLC-ESI-TOFMS-Based Metabolomics and Gene Expression Dynamics Inspector Self-Organizing Metabolomic Maps as Tools for Understanding the Cellular Response to Ionizing Radiation. Anal. Chem. 80, 665–674. 10.1021/ac701807v 18173289 PMC2254319

[B31] Qin J.-X. Yang X.-G. Lv C.-F. Li Y.-Z. Liu K.-K. Zang J.-H. (2021). Nanodiamonds: Synthesis, Properties, and Applications in Nanomedicine. Mater. Des. 210, 110091. 10.1016/j.matdes.2021.110091

[B32] Refsum H. Ueland P. M. Nygård O. Vollset S. E. (1998). Homocysteine and Cardiovascular Disease. Annu. Rev. Med. 49, 31–62. 10.1146/annurev.med.49.1.31 9509248

[B33] Richie J. P. Skowronski L. Abraham P. Leutzinger Y. (1996). Blood Glutathione Concentrations in a Large-Scale Human Study. Clin. Chem. 42, 64–70. 8565235

[B34] Shenderova O. Koscheev A. Zaripov N. Petrov I. Skryabin Y. Detkov P. (2011). Surface Chemistry and Properties of Ozone-Purified Detonation Nanodiamonds. J. Phys. Chem. C 115, 9827–9837. 10.1021/jp1102466

[B35] Shimkunas R. A. Robinson E. Lam R. Lu S. Xu X. Zhang X.-Q. (2009). Nanodiamond-insulin Complexes as pH-dependent Protein Delivery Vehicles. Biomaterials 30, 5720–5728. 10.1016/j.biomaterials.2009.07.004 19635632

[B36] Song Y. Wang X. Zhao C. Qu K. Ren J. Qu X. (2010). Label-free Colorimetric Detection of Single Nucleotide Polymorphism by Using Single-Walled Carbon Nanotube Intrinsic Peroxidase-like Activity. Chem. Eur. J. 16, 3617–3621. 10.1002/chem.200902643 20191629

[B37] Su L.-J. Lin H.-H. Wu M.-S. Pan L. Yadav K. Hsu H.-H. (2019). Intracellular Delivery of Luciferase with Fluorescent Nanodiamonds for Dual-Modality Imaging of Human Stem Cells. Bioconjug. Chem. 30, 2228–2237. 10.1021/acs.bioconjchem.9b00458 31268690

[B38] Sun H. Zhao A. Gao N. Li K. Ren J. Qu X. (2015). Deciphering a Nanocarbon-Based Artificial Peroxidase: Chemical Identification of the Catalytically Active and Substrate-Binding Sites on Graphene Quantum Dots. Angew. Chem. Int. Ed. 54, 7176–7180. 10.1002/anie.201500626 25940927

[B39] Wang H. Li P. Yu D. Zhang Y. Wang Z. Liu C. (2018a). Unraveling the Enzymatic Activity of Oxygenated Carbon Nanotubes and Their Application in the Treatment of Bacterial Infections. Nano Lett. 18, 3344–3351. 10.1021/acs.nanolett.7b05095 29763562

[B40] Wang Q. Chen J. Zhang H. Wu W. Zhang Z. Dong S. (2018b). Porous Co3O4 Nanoplates with pH-Switchable Peroxidase- and Catalase-like Activity. Nanoscale 10, 19140–19146. 10.1039/c8nr06162a 30302476

[B41] Wang Q. Zhang L. Shang C. Zhang Z. Dong S. (2016). Triple-enzyme Mimetic Activity of Nickel-Palladium Hollow Nanoparticles and Their Application in Colorimetric Biosensing of Glucose. Chem. Commun. 52, 5410–5413. 10.1039/c6cc00194g 27009927

[B42] Wang Q. Zhang X. Huang L. Zhang Z. Dong S. (2017). One-Pot Synthesis of Fe3O4 Nanoparticle Loaded 3D Porous Graphene Nanocomposites with Enhanced Nanozyme Activity for Glucose Detection. ACS Appl. Mater. Inter. 9, 7465–7471. 10.1021/acsami.6b16034 28125774

[B43] Wehling J. Dringen R. Zare R. N. Maas M. Rezwan K. (2014). Bactericidal Activity of Partially Oxidized Nanodiamonds. Acs Nano 8, 6475–6483. 10.1021/nn502230m 24861876

[B44] Xia X. Zhang J. Lu N. Kim M. J. Ghale K. Xu Y. (2015). Pd-Ir Core-Shell Nanocubes: A Type of Highly Efficient and Versatile Peroxidase Mimic. Acs Nano 9, 9994–10004. 10.1021/acsnano.5b03525 26333816

[B45] Xing Y. Dai L. (2009). Nanodiamonds for Nanomedicine. Nanomedicine 4, 207–218. 10.2217/17435889.4.2.207 19193186

[B46] Xu Y. Chen X. Chai R. Xing C. Li H. Yin X.-B. (2016). A Magnetic/fluorometric Bimodal Sensor Based on a Carbon Dots-MnO2platform for Glutathione Detection. Nanoscale 8, 13414–13421. 10.1039/c6nr03129c 27346713

[B47] Yan X. Song Y. Zhu C. Song J. Du D. Su X. (2016). Graphene Quantum Dot-MnO2 Nanosheet Based Optical Sensing Platform: A Sensitive Fluorescence "Turn Off-On" Nanosensor for Glutathione Detection and Intracellular Imaging. ACS Appl. Mater. Inter. 8, 21990–21996. 10.1021/acsami.6b05465 27494553

[B48] Zhang L. Han L. Hu P. Wang L. Dong S. (2013). TiO2 Nanotube Arrays: Intrinsic Peroxidase Mimetics. Chem. Commun. 49, 10480–10482. 10.1039/c3cc46163g 24084751

[B49] Zhang S. Ong C.-N. Shen H.-M. (2004). Critical Roles of Intracellular Thiols and Calcium in Parthenolide-Induced Apoptosis in Human Colorectal Cancer Cells. Cancer Lett. 208, 143–153. 10.1016/j.canlet.2003.11.028 15142672

[B50] Zhang X. Hu W. Li J. Tao L. Wei Y. (2012). A Comparative Study of Cellular Uptake and Cytotoxicity of Multi-Walled Carbon Nanotubes, Graphene Oxide, and Nanodiamond. Toxicol. Res. 1, 62–68. 10.1039/c2tx20006f

[B51] Zheng J. Ma L. Xin B. Olah T. Humphreys W. G. Zhu M. (2007). Screening and Identification of GSH-Trapped Reactive Metabolites Using Hybrid Triple Quadruple Linear Ion Trap Mass Spectrometry. Chem. Res. Toxicol. 20, 757–766. 10.1021/tx600277y 17402749

