# Supplementary material for: Single-Atom Fe-Anchored Nano-Diamond With Enhanced Dual-Enzyme Mimicking Performance for H2O2 and Glutathione Detection
Source: Front Bioeng Biotechnol. 2022 Jan 3;9:790849. doi: 10.3389/fbioe.2021.790849 (PMC8762219; doi:10.3389/fbioe.2021.790849)
Supplement: Supplementary file 1 [file DataSheet1.docx]

**Figure S1.** XRD pattern of NDs. All reflection peaks can be indexed to ND.





**Figure S2.** SEM images of Fe-ND


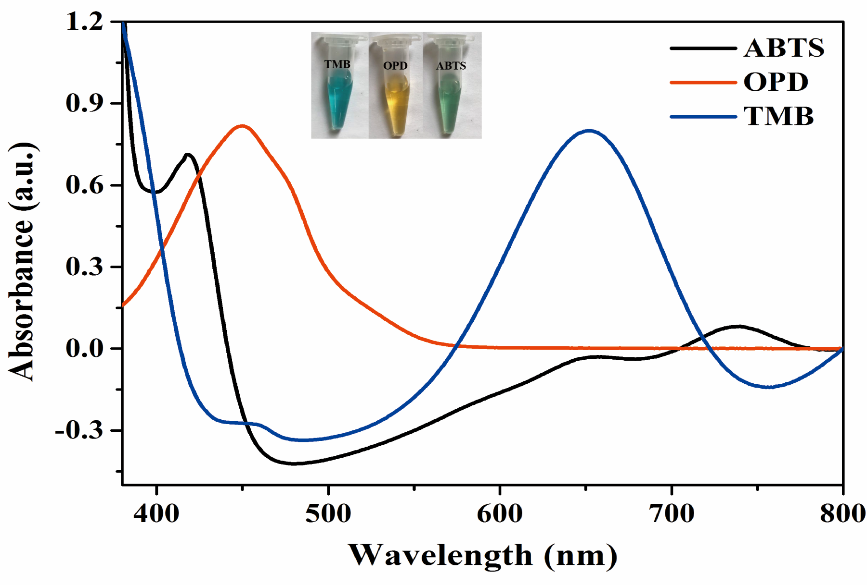


**Figure S3.** Fe-NDs catalyze oxidation of various substrates to produce different color reactions in the presence of H_2_O_2_. (1) ABTS, (2) OPD, (3) TMB.


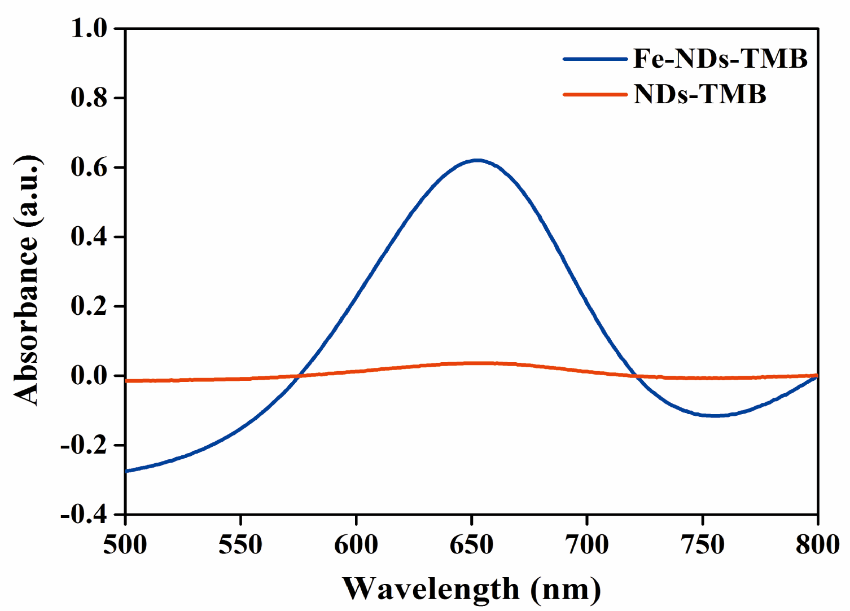


**Figure S4.** The comparison of absorbance of Fe-NDs and NDs at 652 nm.


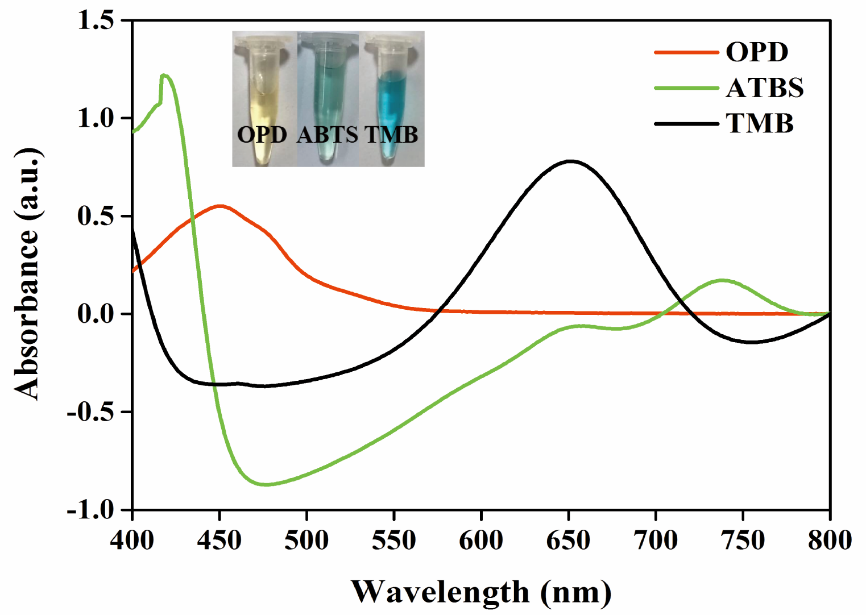


**Figure S5.** Fe-NDs catalyze oxidation of various substrates to produce different color reactions in the absence of H_2_O_2_. (1) ABTS, (2) OPD, (3) TMB.

**Table S1**. Comparison of the *K*_m_ and *V*_max_ values with other peroxidase mimetics.

| Catalysts | *K*_m_ (mM) | | *V*_max_ (10^-8^ M s^-1^) | | Refs. |
| --- | --- | --- | --- | --- | --- |
|  | H_2_O_2_ | TMB | H_2_O_2_ | TMB |  |
| HRP | 3.7 | 0.434 | 8.71 | 10 | Gao et al. (2007) |
| H-GNs | 2.256 | 5.100 | 5.06 | 4.55 | Guo et al. (2011) |
| CoFe_2_O_4_ | 8.89 | 0.387 | 1.93 | 2.90 | Wu et al. (2018) |
| Co_2_V_2_O_7_ | 0.67 | 0.331 | 1.25 | 2.58 | Zhang et al. (2020) |
| Pt-MoO_3_ hybrid | 3.2 | 0.106 | 3.794 | 4.286 | Wang et al. (2014) |
| Fe-NDs | 0.87 | 0.76 | 3.76 | 2.27 | This work |


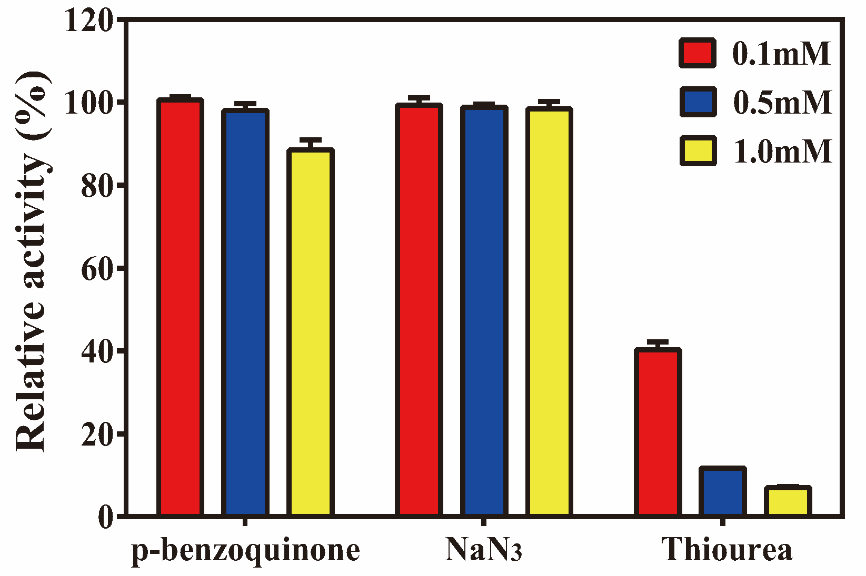

**Figure S6.** Effects of different concentrations of free radical traps on the POD-like activity of Fe-NDs. The error bars are the standard deviation of the third parallel sample.

**Table S2**. Comparison of the *K*_m_ and V_max_ values with other oxidase mimetics.

| Materials | *K*_m_ (mM) | *V*_max_ (10^-8^ M s^-1^) | Refs. |
| --- | --- | --- | --- |
| CeO_2_ NPs | 3.8 | 70 | Asati et al. (2009) |
| Pt NCs | 0.63 | 270 | Yu et al. (2014) |
| NiCo_2_O_4_ MS | 0.127 | 0.999 | Su et al. (2017) |
| Fe-NDs | 0.55 | 4.01 | This work |


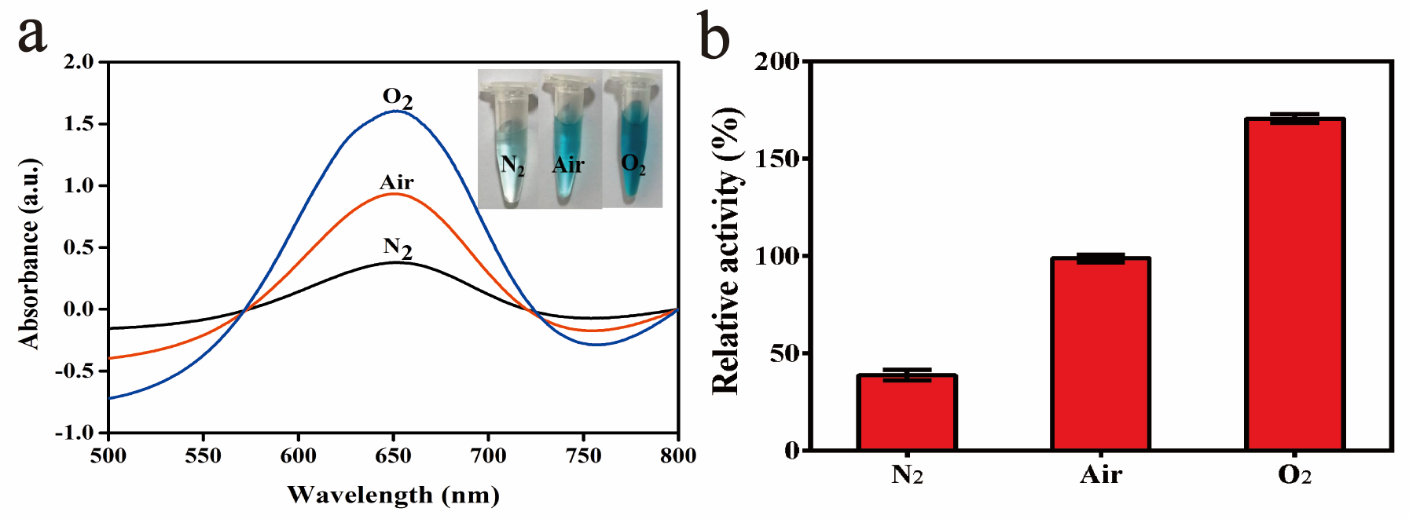


**Figure S7.** (a) Effect of dissolved oxygen on Fe-NDs +TMB system; (b) the effect of N_2_, air and O_2_ on the catalytic activity of Fe-NDs +TMB system. The error bars are the standard deviation of the third parallel sample.


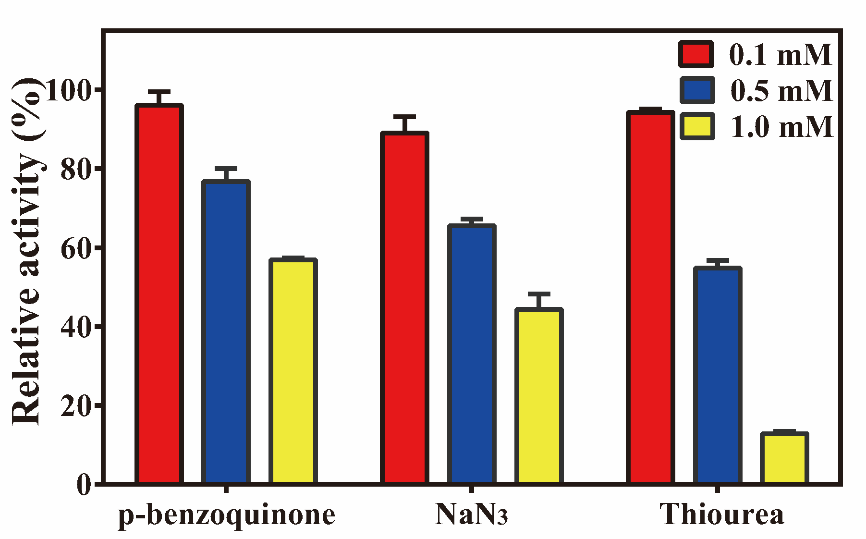


**Figure S8.** Effects of different concentrations of free radical traps on the OXD-like activity of Fe-NDs. The error bars are the standard deviation of the third parallel sample.

**Table S3.** Comparision of different sensors for H_2_O_2_ determination.

| Materials | Linear rang(μM) | LOD (μM) | Method | Refs. |
| --- | --- | --- | --- | --- |
| CoFe | 1-40 | 0.4 | colorimetry | Zhang et al. (2012) |
| Co-Al LDH | 10-50 | 10 | colorimetry | Chen et al. (2013) |
| BiOBr | 0.5-30 | 0.3 | colorimetry | Li et al. (2014) |
| Fe_3_O_4_@MSN | 1-100 | 1 | colorimetry | Wang et al. (2015) |
| CeO_2_ NPs | 0.6-1.5 | 0.5 | colorimetry | Jiao et al. (2012) |
| Fe-NDs | 1-60 | 0.3 | colorimetry | This work |


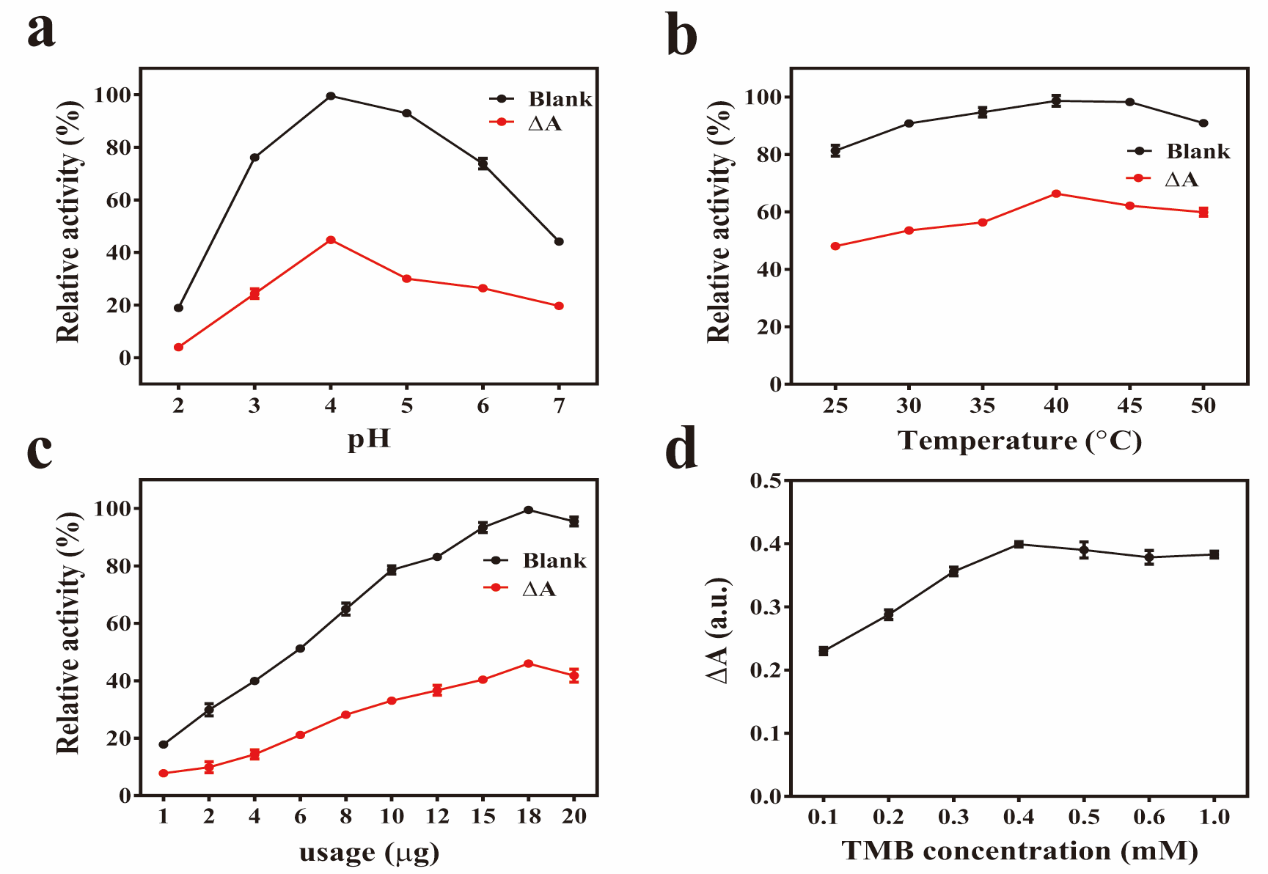


**Figure S9.** Effects of (a) pH, (b) temperature, (c) catalyst dosage, (d) TMB concentration on the catalytic activity of Fe-NDs in the presence of GSH. The error bars are the standard deviation of the third parallel sample.

**Table S4.** Comparision of different sensors for GSH determination.

| Materials | Linear rang (μM) | LOD (μM) | Method | Refs. |
| --- | --- | --- | --- | --- |
| BSA–MnO_2_ NPs | 0.26-26 | 0.1 | colorimetry | Liu et al. (2013) |
| Au NCs | 2-25 | 0.42 | colorimetry | Feng et al. (2017) |
| Fe_3_O_4_ NPs | 3-30 | 3 | colorimetry | Ma et al. (2011) |
| Manganese dioxide nanosheets | 0.5-10 | 0.1 | colorimetry | Huang et al. (2017) |
| MnO_2_ nanosheets | 1-25 | 0.3 | colorimetry | Liu et al. (2017) |
| Co_3_O_4_-Montmorillonite nanocomposites | 0.1-20 | 0.088 | colorimetry | Gao et al. (2018) |
| Fe-NDs | 1-25 | 0.072 | colorimetry | This work |


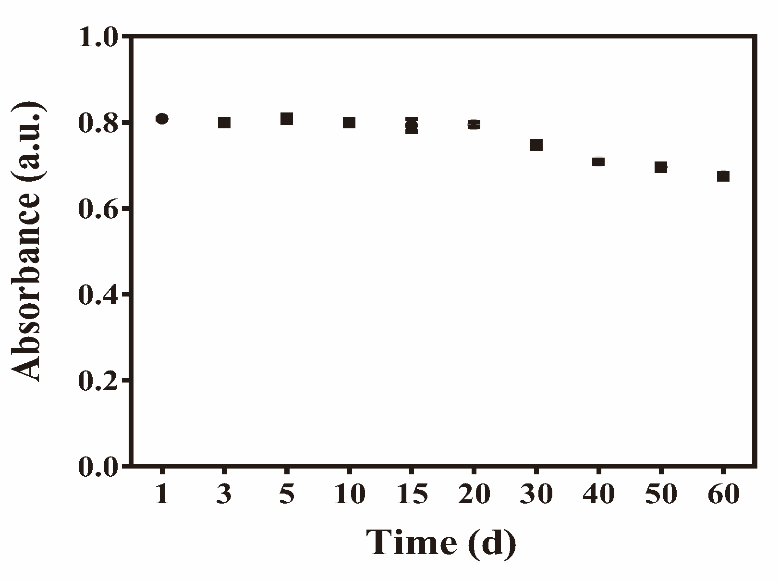


**Figure S10.** The stability of oxidase-like catalytic activity of Fe-NDs. The error bars are the standard deviation of the third parallel sample.

**Table S5**. Comparison of GSH content in medicine detected by HPLC and Fe-NDs

| Analytes | The detection results of HPLC | The detection results  of Fe-NDs |
| --- | --- | --- |
| Tablets (21040750) | 99.86 mg/100.00 mg | 101.74 mg/100.00 mg |
| Injection (20231800) | 0.58 g/0.60 g | 0.59 g/0.60 g |

**References**

Asati, A., Santra, S., C. Kaittanis, S., Nath, S., Perez, M. (2009). Oxidase-Like Activity of Polymer-Coated Cerium Oxide Nanoparticles. Angewandte Chemie. 48, 2308-2312. doi: 10.1002/ange.200805279

Chen, L. J., Sun, B., Wang, X. D., Qiao, F. M., Ai, S. Y. (2013). 2D ultrathin nanosheets of Co–Al layered double hydroxides prepared in L-asparagine solution: enhanced peroxidase-like activity and colorimetric detection of glucose. Journal of Materials Chemistry B. 1, 2268-2274. doi: 10.1039/c3tb00044c

Feng, J. Y., Huang, P. C., Shi, S. Z., Deng, K.-Y., Wu, F. -Y. (2017). Colorimetric detection of glutathione in cells based on peroxidase-like activity of gold nanoclusters: A promising powerful tool for identifying cancer cells. Analytica Chimica Acta. 967, 64-69. doi: 10.1016/j.aca.2017.02.025

Gao, L. Z., Zhuang, J., Nie, L., Zhang, J.B., Zhang, Y., Gu, N., et al. (2007). Intrinsic peroxidase-like activity of ferromagnetic nanoparticles. Nature Nanotechnology. 2, 577-583. doi:10.1038/nnano.2007.260

Gao, Y., Wu, K., Li, H., Chen, W., Fu, M., Yue, K., et al. (2018). Glutathione detection based on peroxidase-like activity of Co_3_O_4_-Montmorillonite nanocomposites. Sensors and Actuators B: Chemical. 273, 1635-1639. doi: 10.1016/j.snb.2018.07.091

Guo, Y. J., Deng, L., Li, J., Guo, S. J., Wang, E., Dong, S. J. (2011). Hemin-Graphene Hybrid Nanosheets with Intrinsic Peroxidase-like Activity for Label-free Colorimetric Detection of Single-Nucleotide Polymorphism. ACS Nano. 5, 1282-1290. doi: 10.1021/nn1029586

Huang, Z.-M., Cai, Q.-Y., Ding, D.- C., Ge, J., Hu, Y.-L., Yang, J., et al. (2017). A facile label-free colorimetric method for highly sensitive glutathione detection by using manganese dioxide nanosheets. Sensors Actuators B: Chemical. 242, 355-361. doi: 10.1016/j.snb.2016.11.066

Jiao, X., Song, H. J., Zhao, H. H., Bai, W., Zhang, L. C., Lv, Y. (2012). Well-redispersed ceria nanoparticles: Promising peroxidase mimetics for H_2_O_2_ and glucose detection. Analytical methods. 4, 3261-3267. doi: 10.1039/c2ay25511a

Li, L. L., Ai, L, H., Zhang, C. H., Jiang, J. (2014). Hierarchical {001-faceted BiOBr microspheres as a novel biomimetic catalyst: dark catalysis towards colorimetric biosensing and pollutant degradation. Nanoscale. 6, 4627-34. doi: 10.1039/c3nr06533b

Liu, J., Meng, L. J., Fei, Z. F., Dyson, P. J., Jing, X. N., Liu, X. (2017). MnO_2_ nanosheets as an artificial enzyme to mimic oxidase for rapid and sensitive detection of glutathione. Biosensors Bioelectronics. 90, 69-74. doi: 10.1016/j.bios.2016.11.046

Liu, X., Wang, Q., Zhang, Y., Zhang, L. C., Su, Y. Y., Lv, Y. (2013). Colorimetric detection of glutathione in human blood serum based on the reduction of oxidized TMB. New Journal of Chemistry. 37, 2174-2178. doi: 10.1039/c3nj40897c

Ma, Y. H., Zhang, Z.Y., Ren, C. L., Liu, G.Y., Chen, X. G. (2011). A novel colorimetric determination of reduced glutathione in A549 cells based on Fe_3_O_4_ magnetic nanoparticles as peroxidase mimetics. Analyst. 137, 485-489. doi: 10.1039/c1an15718c

Su, L., Dong, W. P., Wu, C. K., Gong, Y. J., Zhang, Y., Li, L., et al. (2017). The peroxidase and oxidase-like activity of NiCo_2_O_4_ mesoporous spheres: Mechanistic understanding and colorimetric biosensing. Analytica Chimica Acta. 951, 124-132. doi: 10.1016/j.aca.2016.11.035

Wang, Y. H., Zhou, B., Wu, S., Wang, K. M., He, X. X. (2015). Colorimetric detection of hydrogen peroxide and glucose using the magnetic mesoporous silica nanoparticles. Talanta. 134, 712-717. doi: 10.1016/j.talanta.2014.12.013

Wang, Y. X., Zhang, X., Luo, Z. M., Huang, X., Tan, C. L., Li, H., et al. (2014). Liquid-phase growth of platinum nanoparticles on molybdenum trioxide nanosheets: an enhanced catalyst with intrinsic peroxidase-like catalytic activity. Nanoscale. 6, 12340-12344. doi: 10.1039/C4NR04115A

Wu, L. H., Wan, G. P., Hu, N., He, Z. Y., Shi, S. H., Suo, Y. R., et al. (2018). Synthesis of Porous CoFe_2_O_4_ and Its Application as a Peroxidase Mimetic for Colorimetric Detection of H_2_O_2_ and Organic Pollutant Degradation. Nanomaterials. 8, 451. doi: 10.3390/nano8070451

Yu, C.-J., Chen, T.-H., Jiang, J.-Y., Tseng, W.- L. (2014). Lysozyme-directed synthesis of platinum nanoclusters as a mimic oxidase. Nanoscale. 6, 9618-9624. doi: 10.1039/c3nr06896j

Zhang, X. T., Han, G. J., Zhang, R. Q., Huang, Z., Shen, H., Su, P., et al. (2020). Co_2_V_2_O_7_ Particles with Intrinsic Multienzyme Mimetic Activities as an Effective Bioplatform for Ultrasensitive Fluorometric and Colorimetric Biosensing. ACS Applied Bio Materials. 3,1469-1480. doi: 10.1021/acsabm.9b01107

Zhang, Y. W., Tian, J. Q., Liu, S., Wang, L., Qin, X.Y., Lu, W. B., et al. (2012). Novel application of CoFe layered double hydroxide nanoplates for colorimetric detection of H_2_O_2_ and glucose. Analyst. 137, 1325-1328. doi: 10.1039/C2AN00035K
